# Application of Donabedian quality-of-care framework to assess quality of neonatal resuscitation, its outcome, and associated factors among resuscitated newborns at public hospitals of East Wollega zone, Oromia, Western Ethiopia, 2021

**DOI:** 10.1186/s12887-022-03638-y

**Published:** 2022-10-18

**Authors:** Nuredin Mohammed Guta

**Affiliations:** Department of Nursing, College of Medicine and Health Sciences, Mizan Tepi University, Mizan Aman, Ethiopia

**Keywords:** Neonatal resuscitation, Birth asphyxia, Outcome, East Wollega, Nekemte, Ethiopia

## Abstract

**Background:**

Globally more than 650,000 newborns died on their first day of life from birth asphyxia. The outcome of an asphyxiated newborn depends on the quality of care they received at birth. However, the quality of care newborns received at birth may be below the WHO resuscitation standard. The reason for the poor quality of care is unclear. The Donabedian model, according to the World Health Organization (WHO), is an appropriate framework for health care assessment that focuses on improving the quality of care. So this study aims to assess the quality of neonatal resuscitation, outcome, and its associated factors among newborns with birth asphyxia at public hospitals in the East Wollega zone, 2021.

**Methods:**

An institution-based cross-sectional study was conducted to observe 410 asphyxiated newborns using consecutive sampling methods. Data was gathered using a pretested structured questionnaire and checklist. Epi-data version 3.1 was used to enter data, which was subsequently exported to Statistical Package for Social Sciences version 25 for analysis. A logistic regression model was employed to determine the variables associated with the outcome variable. The adjusted odds ratio with a 95% confidence interval was calculated, and all variables associated with the outcome variable with a *p*-value less than 0.05 in multivariable analysis were determined to be significant factors for the outcome of resuscitated newborns.

**Result:**

A total of 410 asphyxiated newborns were included in this study with a response rate of 97%. From this 87.6% of resuscitated newborns survived. Keeping baby warm [AOR = 6.9; CI (3.1, 15.6)] is associated significantly with increased chances of survival while meconium presence in the airway [AOR = 0.26; CI (0.1, 0.6)], prematurity [AOR = 0.24; CI (0.12, 0.48)], and presence of cord prolapse [AOR = 0.08; CI (0.03, 0.19)] were factors significantly associated with decreased survival of resuscitated newborns at 1 h of life.

**Conclusion:**

Newborns who were kept warm were more likely to survive compared to their counterparts. While the presence of meconium in the airway, preterm, and cord prolapse was associated with the decreased survival status of newborns. Facilitating referral linkage in the event of cord prolapse, counseling on early antenatal care initiation to decrease adverse outcomes (prematurity), and neonatal resuscitation refresher training is strongly recommended.

## Introduction

Worldwide, neonatal deaths account for about 4 million deaths with 99% of these happening in low- and middle-income nations [1, 2]. Furthermore, newborn mortality is still a major problem in most low- and middle-income countries (LMICs), with around 38% of all cases emerging from Sub-Saharan African countries. Ethiopia is one of the five countries responsible for half of the 2.6 million neonatal deaths worldwide in 2019. Birth asphyxia was responsible for about a quarter of these neonatal deaths [3–5]. Even though the mortality and morbidity from birth asphyxia may be preventable and treatable, birth asphyxia-related mortality still accounts for approximately quarter of neonatal mortality worldwide [6, 7].

Neonatal resuscitation is a set of techniques and life-saving interventions used to help newborns establish breathing and circulation at birth. Neonatal resuscitation includes different steps of establishing an airway, breathing, and sometimes circulation in the newborn with breathing difficulty [8, 9]. Simple investments in newborn resuscitation equipment supply and distribution, having Helping Babies Breathe (HBB) training is an important step to improve the survival of these babies from birth asphyxia [10–12]. It is anticipated that providing evidence-based service for all mothers and infant at the moment of birth in facilities may avert up to 531,000 stillbirths, 113,000 maternal deaths, and 1.3 million newborn deaths [13, 14].

Every year, 30 million infants require special or intensive newborn care in a hospital setting, requiring high-quality treatment at the appropriate time and in the right place to survive and thrive as members of society [14]. Resuscitation which is not performed in a timely and effective manner may be responsible for half of the 1.16 million neonatal deaths [15, 16]. Evidence from developing nations reveals that every 30 seconds delay in starting ventilation for up to the first 6 min increases the risk of death by 16%, and every minute delay in applying bag and mask ventilation (BMV) increases the risk of death by 6% [17]. To combat these problems high-quality resuscitation is very important [18, 19]. However, deaths from birth asphyxia occur due to a continuing lack of access to appropriate care in the hospital setting [19]. Also, research in low-income countries demonstrates that a high rate of infant mortality results from poor resuscitation given at birth [20].

Factors such as keeping the baby warm, suctioning before stimulation, mask ventilation, the ability to follow up labour properly, preparing equipment before delivery, with avoidance of meconium or prematurity, are the most effective factors associated with the survival of newborns [11, 21–23].

Even though effective neonatal resuscitation (NR) care has a role in reducing neonatal mortalities [24] still deaths occur as a result of poor quality of care given at birth [25]. Standardizing evaluations of clinical practices and infant services, who undergo resuscitation is currently needed to ensure quality care [10, 26]. To ensure this aim, many neonatal survival strategies including obstetric care and quality neonatal resuscitation in various contexts were established [5, 6, 27]. However, neonates with birth asphyxia still had a low survival rate, especially in a low resource setting [28]. Improved routine data collection focusing on quality of resuscitation and outcomes is required to accelerate progress [14]. The Donabedian model is one of the models recommended to assess the quality of care by WHO [29]. Three components of the model were used to assess the elements of quality of intrapartum care described as structure, process, and outcome [30]. The structure part was the human resource of the hospital, availability of equipment, and process part is neonatal resuscitation performed while survival or death of neonate is as an outcome of the study. So, this study aims to address quality neonatal resuscitation, its outcome, and associated factors by using the Donabedan model among resuscitated newborns with birth asphyxia at public hospitals in the East Wollega zone.

## Methods

### Study setting and design

From March 1 to June 30, an institution-based cross-sectional study was done at Wollega university referral hospitals, Nekemte specialized hospital, Gida Ayana district hospital, Jimma Arjo hospital, and Sire district hospitals in the East Wollega zone. During the study period, all asphyxiated newborns in public hospitals in the East Wollega zone were included, while congenital anomalies incompatible with life, mothers of newborn babies receiving opioids, and other depressive drugs were excluded.

### Sampling and data collection

The sample size was estimated using the single population proportion formula, taking into account the following factors: The population proportion (P) is 50%, the confidence interval is 95%, the margin of error is 5%, and the response rate is 10%. As a result, 422 asphyxiated neonates from all governmental hospitals in the research area were evaluated. The previous 4 months’ data of asphyxiated newborns of all hospitals from the delivery registration book were observed as follows. The delivery registration book at the relevant institutions (Wollega University Referral Hospitals (102), Nekemte Specialized Hospital (121), Gida Ayana (88), Sire (80), and Arjo (85) revealed a total of 476 deliveries. The computed sample size was allocated proportionally for each hospital based on these data. Seventy-eight (78), seventy-five (75), and seventy-one (71) asphyxiated babies were observed from Nekemte specialized hospital, ninety-one (91) from Wollega University referral Hospital, and seventy-eight (78) from Gida Ayana, Arjo, and Sire district hospitals, respectively. Then, until the desired sample size was reached, asphyxiated newborns were observed consecutively.

### Variables of the study

The Donabedian domains of structures and processes were the focus of the elements influencing the infant outcome. “Yes = 1” and “no = 0” were used to construct the independent process variables. The four sections of the NR process were used to categorize process variables. Drying/stimulation (drying, removing wet cloth, and keeping baby warm) airway maintenance (checked airway, suctioning, positioning baby’s head) bag and mask breathing, and possible cardiac resuscitation. NR equipment availability, health care professional characteristics such as training/qualifications and experience, and NR training were used to assess structural factors. The newborn’s survival status at 1 hour was the primary outcome. The outcome was either alive or dead [30, 31]. The outcome variable, survival or death of neonate was recorded starting from delivery time to till one hour post-partum. Newborns who survived past 1 h after delivery were recorded as survived and death was recorded as it was confirmed by physicians immediately after noting the time of death within 1 h of life. Newborns transferred to the neonatal intensive care unit (NICU) within 1 h for further care were evaluated at NICU by a neonatal nurse and death were immediately recorded and reported.

### Study instrument and measurement

The data collection tool was developed and modified from a variety of studies undertaken in developing countries and then contextualized to Ethiopia [23, 32, 33]. The tool was then translated into Afan Oromo, the working language, and then retranslated back to English for consistency. These tools were divided into three parts: socio-demographic characteristics of the provider, a neonatal resuscitation process checklist, and neonatal-related data. First, at the delivery site where neonatal resuscitation is conducted is observed for equipment, and a checklist was used to check the neonatal resuscitation procedure. 10 data collectors, five BSc nurses and five BSc midwives were recruited for data collection, two BSc nurses supervised the total data gathering activity.

### Operational/term definitions

Keeping baby warm is defined as sampling wrapping with cloth after drying and stimulation by placing near radiant heater.

Birth asphyxia in this context was defined as failure to initiate and sustain breathing at birth [16].

Prematurity: newborns with a gestational age of 28 weeks of gestation to 37 weeks of gestation.

Term: newborns with the gestational age of 37 weeks to 42 weeks.

Previous training: Any types of neonatal resuscitation training taken previously past 6 months.

### Data quality control

Before the commencement of data collection for the study, a pilot was conducted in Bako hospital on 5% of the sample size to identify unclear phrasing and any uncertainty in the understanding of data collection instruments. The Chronbach alpha was determined to be 0.76. After the pretest, the Apgar score was modified to one, five, and 10 min. Additionally, data collectors and supervisors provided 2 days of training on how to obtain verbal consent, as well as informed consent from a mother under the age of 18, maintain confidentiality, and respect the rights of participants, as well as study purpose, methodology, how to conduct the interview, and how to observe the resuscitation process, including how to assess the Apgar score. 10 BSc (5 nurses and 5 midwifes) served as data collectors. To reduce bias during observation of neonatal resuscitation procedures, health care professionals were observed by B.Sc. nurses while performing resuscitation, but occasionally recruited midwives working in delivery rooms also collected data to reduce the hawthorn effect, which means that when data collectors were from other sites, they tried to perform resuscitation well because they knew they were under observation. The hawthorn effect was minimized when data collectors were viewed by a member of their team since they perform the technique as standard treatment. Furthermore, data collectors are constantly present in the labor ward to observe health care providers’ usual practices in the delivery room. To ensure the completeness, accuracy, and consistency of information collected during data collection, the supervisors supervise data collectors twice a week, and in the meantime, the principal investigator went to the hospitals and double-checked proper data collection.

### Data analysis

Gathered data was then entered into Epidata version 3.1 before being exported to SPSS version 25 for further analysis. Data were cleaned, edited, categorized, and outliers were removed and prepared to be presented in the form of a table, chart, and percentage before analysis. To find factors that have been associated with the outcome variable, a logistic regression analysis was used. Factors that were associated with the outcome variable at a 25% (*P*-value ≤0.25) significant level in the bivariable analysis were prepared for final multivariable analysis after multicollinearity was checked with variance inflation factors, as well as backward logistic regression analysis was used to control confounding. In addition, model fitness was done by the Hosmer-Lemeshow goodness of fit test with a cut point of 0.5. Adjusted odds ratios (AOR) with 95% confidence intervals were computed and statistical significance was declared when it is significant at a 5% level (*p*-value < 0.05).

## Results

### Structural factors

#### Health care Provider’s background characteristics

A total of 75 health care professionals (HCP), with a mean age of 28.2 years gave care to the newborns. More than half of them, 39 (52%) were men. Midwives were the most common care providers of neonatal resuscitation, accounting for more than two-thirds, 61(84.4%) of the respondents. Nearly half of HCPs, 39 (52%) said they have ever attended NR training (Table 1).

**Table 1:** Background characteristics of HCPs at public hospitals of East Wollega zone, west Oromia, Ethiopia; March –June, 2021

HCP Characteristics	Category	Frequency	Percentage (%)
Age (years)	< 25 years	**14**	**17.1**
	> 25 years	**61**	**82.9**
Sex	Male	**39**	**51.7**
	Female	**26**	**48.3**
Profession	midwife	**61**	**84.4**
	Nurse	**11**	**13.9**
	IESO	**2**	**0.5**
	Specialist	**1**	**0.2**
Previous training in NR	Yes	**39**	**52**
	No	**36**	**47.9**
Period since the last NR	< =1 year	**48**	**64.5**
	> 1 years	**27**	**35.5**
Duration of practice	< 2 year	**34**	**45.7**
	> 2 years	**41**	**54.3**

#### Health facility characteristics

The resuscitation area had guidelines posted. All of the fundamental NR equipment is available in the delivery pack, suction devices such as (electric-powered suction machine and suction bulbs), term face masks (size = 1), and one clean dry towel for cleaning the newborn. The delivery room, on the other hand, lacked a wall clock and a flip chart that showed the procedure clearly.

### Process factors

#### Overall neonatal Resuscitation processes performed

##### Drying or stimulation

The majority of babies, 338 (82.4%), were gently dried by massaging their backs with a dry towel. The wet cloth was removed from 347 (84.6%) of the neonates that were resuscitated, and 365 (89%) of the newborns were wrapped in a cloth to keep them warm.

##### Checked airway

More than two-thirds of newborns who did not respond to airway secretion stimulation had their airways checked and cleared. In 240 (58.5%) of the infants, meconium was found in the airway. In 230 (56%) of neonates with meconium present, proper head positioning to clear the airway was seen.

##### Bag and mask ventilation

Bag and mask ventilation (BMV) (*n* = 164) was initiated for all newborns who did not respond after airway clearance. However, of the babies who received BMV, 128 (78%) started ventilation within the first minute. The median time for BMV to start was 30 seconds (SD =29.2). All newborns who did not respond to initial bag and mask ventilation (*n* = 96) were initiated subsequent BMV.

##### Advanced care

Among newborns who received advanced care (*n* = 57), chest compressions with successful breaths were given to 54 (94.7%) neonates while 39 (68.4%) of them received supplemental oxygen. Adrenaline was administered in 9 (15.9%) newborns who needed advanced resuscitation (Table 2).

**Table 2:** Practice of neonatal resuscitation process at public hospitals of East Wollega zone, Oromia, Western Ethiopia; March –June, 2021

NR process performance	Category	Frequency (***n*** = 410)	Percentage (%)
**Drying/Stimulation(410)**			
Baby dried	Yes	**338**	**82.4**
	No	**72**	**17.6**
Wet cloth removed	Yes	**347**	**84.6**
	No	**63**	**15.4**
Baby kept warm	Yes	**365**	**89**
	No	**45**	**11**
**check/open airway**			
Checked airway(410)	Yes	**376**	**91.7**
	No	**34**	**9.3**
Meconium present	Yes	**240**	**58.5**
	No	**170**	**41.5**
suctioning done before stimulation	Yes	**233**	**56.8**
	No	**177**	**43.2**
Baby’s head in neutral position	Yes	**230**	**56**
	No	**170**	**44**
**BMV initiated (164)**			
Initial BMV initiated within the golden minutes	Yes	**128**	**78**
	No	**36**	**22**
Subsequent BMV(96)	Yes	**83**	**86.5**
	No	**13**	**13.5**
Correct mask size used	Yes	**88**	**91.7**
	No	**8**	**8.3**
Chest movements observed	Yes	**87**	**90.6**
	No	**9**	**9.4**
**Advanced (57)**			
Chest compressions	Yes	**54**	**94.7**
	No	**3**	**5.3**
Supportive oxygen	Yes	**39**	**68.4**
	No	**18**	**31.6**
Adrenaline administered	Yes	**9**	**15.9**
	No	**48**	**84.1**
Final outcome	Survived	**359**	**87.6**
	Died	**51**	**12.4**

### Outcome factors

#### General characteristics of newborns resuscitated

Nearly half of the neonates 222 (54.1%) who were resuscitated were females, with spontaneous vertex delivery (SVD) accounting for 227 (55.4%) of the deliveries. The median birth weight was 3500 g (SD 508.85). While 302 (73.7%) of the babies were over 37 weeks of gestational age. At 1, 5, and 10 minutes, the mean Apgar score was 5.5, 5.9, and 6.8 respectively (Table 3).

**Table 3:** General characteristics of resuscitated newborns at public hospitals of East Wollega zone, Oromia, Western Ethiopia; March –June, 2021

Characteristics	Category	Frequency(***n*** = 410)	Percentage (%)
Mode of delivery	SVD	227	**55.4**
	Operative virginals delivery	141	**34.4**
	C/S	42	**10.2**
Sex	Male	222	**51.4**
	Female	188	**45.9**
Birth weight (grams)	< 2500 g	19	**4.6**
	2500 -4000 g	336	**82**
	> 4000 g	55	**13.4**
Gestational age (weeks)	< 37 weeks	108	**26.3**
	≥ 37 weeks	302	**73.7**
One minutes Apgar score	Less than or equal to three(<=3)	22	**5.36**
	Greater than three(> 3)	388	**94.64**
Five min Apgar score	Less than or equal to three(<=3)	11	**2.68**
	Greater than three(> 3)	399	**97.3**
Ten min Apgar score	Less than or equal to three(<=3)	17	**4.14**
	Greater than three(> 3)	393	**95.86**
Presentation of newborn	Cephalic	358	**87.3**
	None cephalic	52	**12.7**

#### Final outcome of resuscitated newborns

**Fig. 1:**
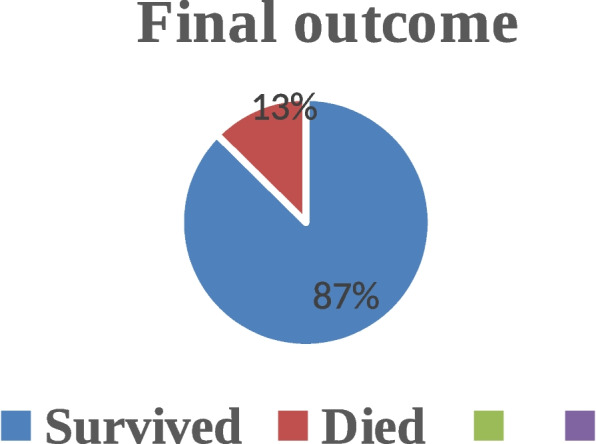
Final neonatal resuscitation outcome among newborns with birth asphyxia at public hospitals of East Wollega zone, Oromia, Western Ethiopia, 2020

The study revealed that among asphyxiated newborns undergoing neonatal resuscitation 359 (87.6%) of them survived the first hour of life (Fig. 1).

#### Factors associated with neonatal resuscitation outcome

Multivariable logistic regression analysis was conducted with those variables with *p* values < 0.25 in bivariable logistic regression analysis. Accordingly keeping the baby warm, absence of meconium in the airway, increased gestational age, and absence of cord prolapse were significantly associated with the survival of the newborn at 1 h of life.

Newborns kept warm (AOR, 95%CI; 6.9(3.1, 15.6)) were 6.9 times more likely to survive in comparison to those not kept warm. Newborns with meconium presence in the airway (AOR, 95%CI; 0.26(0.1,0.6)) had 26 times higher odds of death compared to counter parts. A premature infant (AOR, 95%CI; 0.24(0.12, 0.48)) is 76%less likely to survive compared to a term infant. Also, this study found that newborns with cord prolapse (AOR, 95%CI; 0.08(0.03, 0.19)) were 92% less likely to survive compared to those with no cord prolapse (Table 4).

**Table 4:** Bivariable and Multivariable Analysis of Factors Associated with Neonatal Resuscitation Outcome at Public Hospitals of East Wollega zone, Oromia, Western Ethiopia; March –June, 2021

Factors	Category	NR outcome,*n* = 410		COR(95CI)	AOR(95%CI)	*p*-value
		Survived	Died			
Drying baby	Yes	304 (74.14)	34 (7.81)	2.76 (1.5,5.3)	1.4 (0.6,3.5)	0.47
	No	55 (13.41)	17 (4.15)		1	
Removing wet cloth	Yes	310 (75.6)	37 (8.99)	2.4 (1.2,4.8)	1.5 (0.5,4.1)	0.45
	No	49 (77.7)	14 (22.3)		1	
Baby kept warm	Yes	331 (90.7)	34 (9.3)	5.9 (2.9,11.9)	6.9 (3.1,15.6)	**.001**
	No	28 (66.6)	17 (33.4)	1	1	
Meconium	Yes	201 (83.75)	39 (16.25)	0.4 (0.2,0.8)	0.26 (0.1,0.6)	**0.001**
	No	158 (96.42)	12 (3.46)	1	1	
Gestational age	Preterm	85 (76.9)	23 (23.1)	0.3 (0.14,0.47)	0.24 (0.12,0.48)	**0.001**
	Term	274 (90.7)	28 (9.3)	1	1	
NRFHBP	Yes	127 (79.4)	33 (20.6)	3.4 (1.2,6.2)	0.3 (0.16,0.55)	0.11
	No	232 (92.8)	18 (7.2)	1	1	
Cord prolapse	Yes	17 (49.6)	18 (51.4)	0.09 (0.04,0.20)	0.08 (0.03,0.19)	**0.001**
	No	342 (91.2)	33 (8.8)	1		

Factors are statistically significant at *p* value < 0.05 and all significant values are written in bold letter

*AOR* Adjusted odd ratio, *COR* Crude odd ratio, *NRFHBP* None -reassuring fetal heart beat pattern

## Discussion

The goal of this study was to evaluate the quality of neonatal resuscitation and its outcomes and factors associated with mortality/survival at public hospitals in the East Wollega zone of Oromia, Western Ethiopia, in 2021. Among 410 newborns resuscitated, 359 (87.6%), [CI = 84.1, 90.7] survived the first hour of life. This result was similar to 87.7% survival found in a study conducted in northern Tanzania, [34], and 86.7% in South Africa [35]. Also supported by an 86.2% success rate in an institution-based study in Kenya on the practice and outcome of neonatal resuscitation among newborns with birth asphyxia [11]. However it was higher than a study conducted in Iran which reported 76.6% survival [36], but lower than the studies conducted in Tanzanian rural hospitals (93.6%) [6] and sub-Saharan Africa (90.9%) [37]. This variation may be due to the difference in design and sample size of the study conducted in these countries.

Survival of asphyxiated newborns after resuscitation may be affected by structural factors such as health care professional characteristics (year of experience, and previous training), availability of important equipment in the institution to give care as well as process factors such as the effective implementation of neonatal resuscitation procedure [29, 38, 39].

This study also revealed that some neonatal resuscitation equipment or materials such as availability of resuscitation guidelines (flow chart), and clock were absent in participating resuscitation areas. Absence of such equipment affects quality of resuscitation care given as well as neonatal outcome. Also study findings from low and middle-income countries demonstrate that facilities equipped to give better care to newborns improves the outcome of newborns with difficulty in breathing [40, 41].

Health care professionals performing neonatal resuscitation should be trained and sufficiently skilled to deliver quality care for newborns [40, 42, 43]. This study revealed that nearly half of HCP had no any type of previous training for neonatal resuscitation, nearly half of them had a duration of practice greater than 2 years, and more than one-third had a duration of more than 1 year of current training. Other studies have reported that health care professionals having any type of NR training compared to those not having it all, longer duration of practice, short duration of the training, or having immediate refresher training less than 6 weeks were associated with given better quality resuscitation for asphyxiated newborns [31, 43].

Proper resuscitation practice were linked to improved neonatal outcome. Greater attention to care given has proven to have a beneficial impact on the survival of newborns. Keeping a baby warm [AOR = 6.9; 95% CI (3.1, 15.6)], is one of the factors significantly associated with the survival of newborns at 1 h of life, which was similar to studies conducted in Kenya [11] and Sub-Saharan Africa [44]. This may be because keeping the baby warm reduces the risk of neonates developing hypothermia and is associated with improved survival of newborns. However, this study reports some health care professionals perform stimulation before suctioning. But, suctioning should be done before stimulation in case the airway is obstructed. This health care professional practice also contradicts different resuscitation guidelines [16, 39].

Also, this study reported that BMV was initiated for all newborns who did not respond after airway clearance. Subsequent BMV was continued in more than two-thirds. However, of the babies who needed assistance, one-third did not start ventilation within the first minute after birth. Immediate intervention within the first minute potentially reduces hypoxia and related complications. The practice of health care professionals in the first minute initiation was poor in this study. A delay in the application of BMV is associated with poorer neonatal outcomes due to decreased oxygen supply. This is revealed by several international studies [17, 28].

Among newborns who needed advanced care, chest compressions with successful breaths were given to neonates with supplemental oxygen. Newborns who got advanced care after application of continuing BMV had better outcomes to assist breathing and circulation. If breathing and circulation is not maintained after initial BMV, the newborn may end up with severe asphyxia and related complications [39]. However, this study reported that the majority of the newborn’s received chest compression rather than receiving subsequent BMV to support ventilation. This is contrary to different resuscitation guidelines [16, 39]. This misapplication of skills may worsen rather than improving the outcome. This may be due to nearly half of health care professionals in the study area having no training in neonatal resuscitation.

This study found that Premature infants (gestational age less than 37 weeks) [AOR = 0.24; 95% CI (0.12, 0.48)] were 76% less likely to survive than those with a gestational age of greater than 37 weeks. This is supported by the study conducted in Gonder [45], and Tanzania [6]. But contradicts studies from South Africa [35] and Nigeria [46]. This discrepancy may be due to differences in the care of premature newborns or the different setup of resuscitation areas.

The present study also found that newborns with the presence of meconium-stained liquor [AOR = 0.26; 95% CI (0.12, 0.57)], in the airway had reduced chances of survival within the first hour of life. This agreed with outcomes reported in Kenya [11], South Africa [35], India [47], and brazil [48]. This is may be due to meconium presence in the airway blocking air entry which leads to blockage of the airway resulting in the death of newborns.

The study found that newborns with Cord prolapse [AOR = 0.08; 95% CI (0.03, 0.19)] had 92% less likely to survive compared to those with no cord prolapse. Another study also reported cord prolapse as an independent risk factor for neonatal mortality [22]. The possible reason will be due to interference in blood supply to newborns while delivery which in turn decreases the oxygen supply to newborns resulting in hypoxia. The present study shares limitations of the cross-sectional study [22]., as well as limitations of the Donabedian model in which sociodemography is not considered as the main variable. The study also measures the survival of neonates at 1 h. But several studies measures neonatal mortality at 24 hours, 7 days and at 1 month. So this study may underestimate actual newborn survival.

## Conclusion

At 1 h after being resuscitated, 87.6% of resuscitated neonates survived birth asphyxia. Keeping the baby warm was a factor that was associated with increased chances of survival while meconium presence in the airway, being premature, and having cord prolapse were all associated with a decreased chance of survival from birth asphyxia within the first hour of life. In comparison to other research, survival of resuscitated newborns is still poor after a short length of time. Health practitioners working in antenatal care clinics and labor and delivery wards should receive training to improve their resuscitation skills as well as the ability to recognize and detect early problems such as cord prolapse that affect the prognosis of resuscitated neonates. To intervene in cases of cord prolapse, strengthen formal referral links with peripheral health services. To avoid missing the neonatal resuscitation procedure steps, focus on following the neonatal resuscitation process guideline and charts accessible in resuscitation areas. More research on newborn resuscitation outcomes is needed, with a case-control study design being used to measure better neonatal resuscitation outcomes.

## Data Availability

All data generated or analyzed during this study are included in this published article.

## Abbreviations

EDHS: Ethiopian Demographic Health Survey
FDRE: Federal Democratic Republic of Ethiopia
FANC: Focused Antenatal Care
HBB: Helping Baby Breath
HCP: Health Care Professional
IMNCI: Integrated Management of Newborn and Child Hood Illness
MCNH: Maternal Child and Neonatal Health
MAS: Meconium Aspiration Syndrome
NICU: Neonatal Intensive Care Unit
NRFHRP: None Reassuring Fetal Heart Rate pattern
PPV: Positive pressure ventilation
SPSS: Statistical Package for Social Science
WHO: World Health Organization
